# Detection of Severe Neurological Complications Caused by the Removal of Central Venous Catheter Accidentally Inserted in the Epidural Space via Motor-Evoked Potential: A Case Report

**DOI:** 10.7759/cureus.71435

**Published:** 2024-10-14

**Authors:** Tokimitsu Hibino, Yusuke Okui, Satoko Kondo, Fumiko Ogura, Yoshie Toba

**Affiliations:** 1 Department of Anesthesiology, Seirei Hamamatsu General Hospital, Hamamatsu, JPN

**Keywords:** anesthesia, angiography, central venous catheter (cvc), decompressive laminectomy, motor-evoked potential

## Abstract

The central venous catheter (CVC) has been in clinical use for more than half a century. It was initially used for total parenteral nutrition. However, its indication gradually expanded to chemotherapy, intensive care, anesthesia, and other areas. As the application of CVCs increased, complications also increased. Nevertheless, some guidelines for CVC insertion have been implemented, and clinicians worldwide are working hard to prevent complications during CVC insertion. However, the safety of CVC removal has not been given adequate attention. Because of a few reports on complications, such as air embolism and airway obstruction, clinicians are recognizing the potential risks associated with CVC. However, a few medical staff recognize the possibility of associated neurological complications.

We herein report a case of a patient who underwent anesthesia for the removal of a CVC, which was inadvertently inserted in the epidural space. The catheter was used to monitor central venous pressure and as a route for medicine administration before the recognition of its abnormal position. Although the distal luminal wave pattern was similar to that of a normal central venous line, heparin did not exert its expected effect after administration from the distal lumen. Conversely, appropriate blood pressure responses were observed following the administration of inotropic agents from the proximal lumen. Objective neurological monitoring was required for removal because of the involvement of general anesthesia. After general anesthesia induction, the surrounding tissue of the CVC was dissected toward the deep layer of the neck. Arterial bleeding occurred immediately after removal. After 33 minutes, the motor-evoked potential (MEP) waves deteriorated. Angiography showed bleeding from the left vertebral artery into the spinal canal. Consequently, emergency coil embolization of the left vertebral artery was performed, followed by emergency laminectomy to decompress the spinal canal. All procedures were completed, and the MEP waves completely recovered. The postoperative course was uneventful, and the patient was discharged after 17 days. In this case report, we discuss the appropriate removal steps for a CVC inadvertently placed in the epidural space.

## Introduction

The central venous catheter (CVC) was first inserted in 1929 when a 25-year-old German surgical resident, Werner Forssmann, punctured his left antecubital vein and passed a 4 Fr ureteric catheter through his right atrium [1]. At that time, the German Medical Association rejected his work, and he was fired from his position. However, CVC was gradually recognized as an important tool. In 1945, the general concept of total parenteral nutrition in pediatric patients was advocated [1]. Dudrick et al. brought up six puppies to mature dogs via total parenteral nutrition using CVC in 1968 [2]. Subsequently, CVC has been widely accepted and used for long-term total parenteral nutrition, and its indications have expanded.

Currently, approximately 15 million CVCs are used annually in the United States for chemotherapy, intensive care, and anesthesia management [1]. With the increase in CVC applications, adverse events have also increased. Several guidelines have been implemented for safe CVC insertion, and medical staff are attempting to prevent associated complications [3,4]. Previously, little attention had been paid to the safety of CVC removal, and the task was assigned to junior members of the surgical team [5]. Adverse events related to CVC removal, such as air embolism and airway obstruction [6,7], have been reported, leading to the gradual recognition of potential risks. Drewett comprehensively described the adverse events of CVC extraction and methods of preventing them [8] but did not discuss the neurological complications following CVC extraction, as their report was based on the premise that the CVC was inserted correctly. However, there are only a few reports on neurological complications after extraction of an inappropriately inserted CVC, resulting in arterial injury or placement in the epidural space [9]. In the present case, anesthesia was used for the removal of a CVC that had been inadvertently placed in the epidural space. Neurological evaluation was performed using motor-evoked potentials (MEPs). Following CVC removal, the MEP waveforms gradually attenuated and eventually flattened, indicating that they were due to arterial hemorrhage and acute spinal canal compression caused by CVC-induced arterial damage. Emergency coil embolization of the injured artery and laminectomy allowed recovery without sequelae. We discuss an appropriate strategy for extracting misplaced CVC and the need for neurological evaluation.

## Case presentation

**Figure 1:**
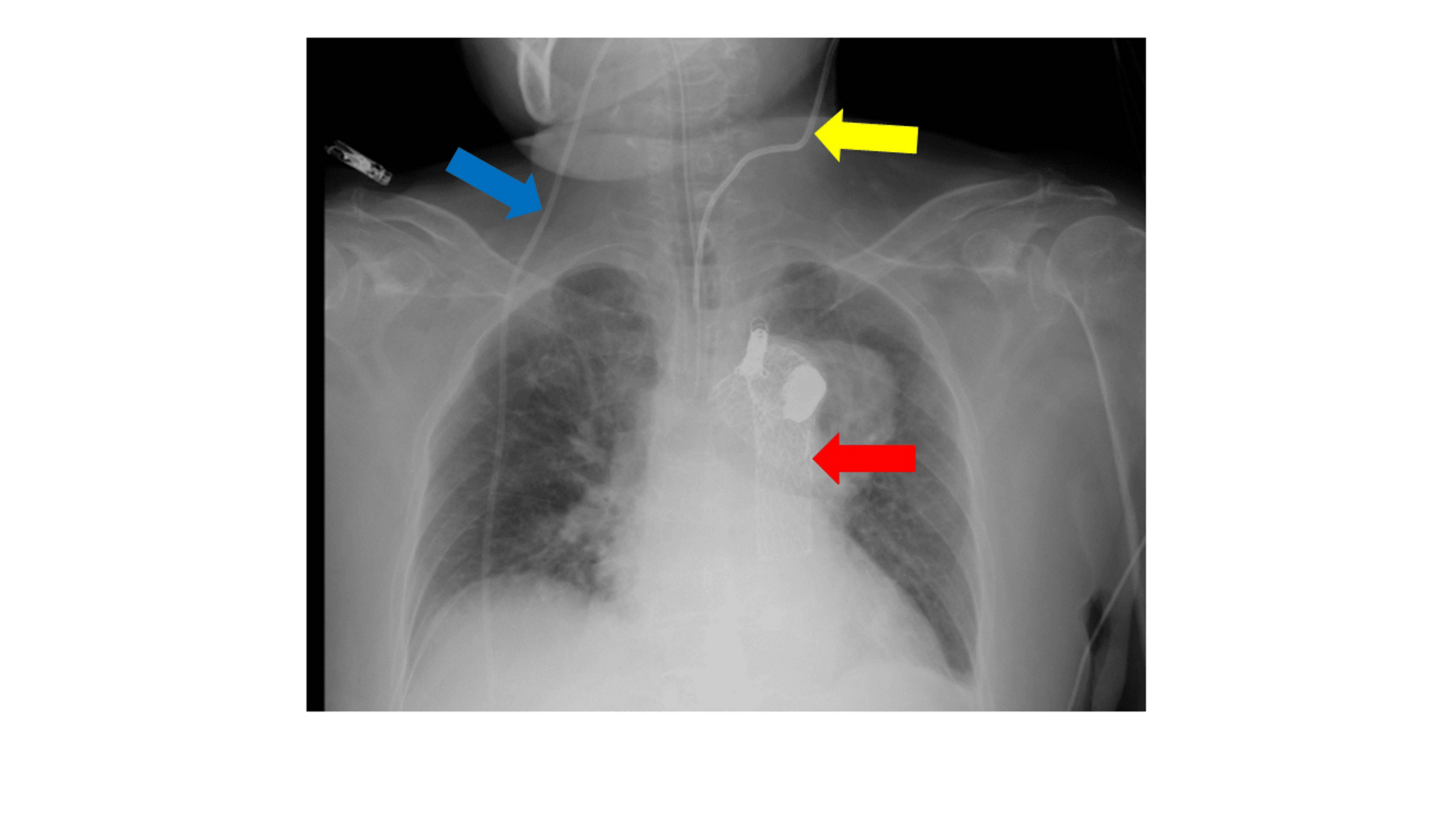
Chest radiograph after stenting for thoracic aortic aneurysm The yellow arrow indicates the central venous catheter, the red arrow indicates the thoracic stent graft, and the blue arrow indicates the ventriculoperitoneal shunt. The central venous catheter inserted from the left internal jugular vein did not enter the innominate vein into the superior vena cava but was descending the midline.

**Figure 2:**
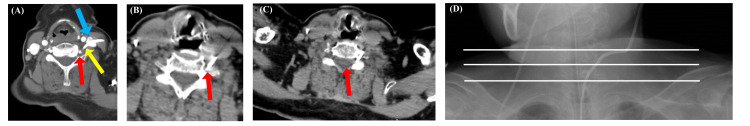
Computed tomography images showing the central venous catheter going through the left internal jugular vein and entering the spinal cord through the C6/C7 intervertebral foramen CVC: Central venous catheter; C6: sixth cervical vertebra; C7: seventh cervical vertebra Computed tomography images show that the CVC is going through the left internal jugular vein and entering the spinal cord through the C6/C7 intervertebral foramen. (A) The yellow arrow indicates the CVC, the red arrow indicates the C6/C7 intervertebral foramen, and the blue arrow indicates the left internal jugular vein penetrated by the CVC. (B) The arrow indicates the C6/C7 intervertebral foramen. (C) The arrow indicates the spinal canal. (D) The upper white line indicates the position of the image shown in (A), the middle line indicates the position of the image shown in (B), and the lower line indicates the position of the image shown in (C). A CVC was inserted going through the left internal jugular vein, through the C6/C7 intervertebral foramen and into the spinal canal

An 81-year-old woman underwent thoracic endovascular aortic repair for a thoracic aortic aneurysm. Before surgery, a CVC was inserted under echocardiographic guidance to monitor central venous pressure (CVP) and administer medications during stent insertion. CVP monitoring was performed in the distal catheter lumen, and waveforms resembling the CVP pattern were observed. Heparin, which was administered through the distal lumen, was not effective and required redosing via a peripheral venous line. In contrast, increases and decreases in blood pressure were observed with increasing and reducing catecholamine administration from the proximal lumen, respectively. In total, 11 mL heparin and 5.15 mL dopamine were administered from the distal and proximal lumens, respectively. The CVC location was confirmed postoperatively. A chest X-ray performed after the procedure revealed malposition of the CVC (12 gauge triple lumen Argyle™ Fukuroi SMAC™ plus, Cardinal Health, Dublin, OH) (Figure 1). The CVC was inserted into the left internal jugular vein because of a ventriculoperitoneal shunt that passed through her right neck. The patient was extubated and then examined by computed tomography (CT) for further evaluation of the CVC malposition. The CT image showed that the CVC went through the left internal jugular vein, passed through the intervertebral foramen between the 6th and 7th cervical vertebrae, and reached the epidural space (Figure 2). On CT images, the CVC appeared to be in contact with the left vertebral artery, and it was not clear if it was actually damaged. Therefore, there was a concern about whether the CVC injured the left vertebral artery. CT revealed that the contrast smoothly flowed through the left vertebral artery; thus, the flow of this artery appeared to have been maintained. However, it was unclear whether the left vertebral artery was injured. A conference involving anesthesiologists, cardiovascular surgeons, otorhinolaryngologists, neurosurgeons (mainly engaging in neurovascular therapy), and orthopedic surgeons was immediately held. 

**Figure 3:**
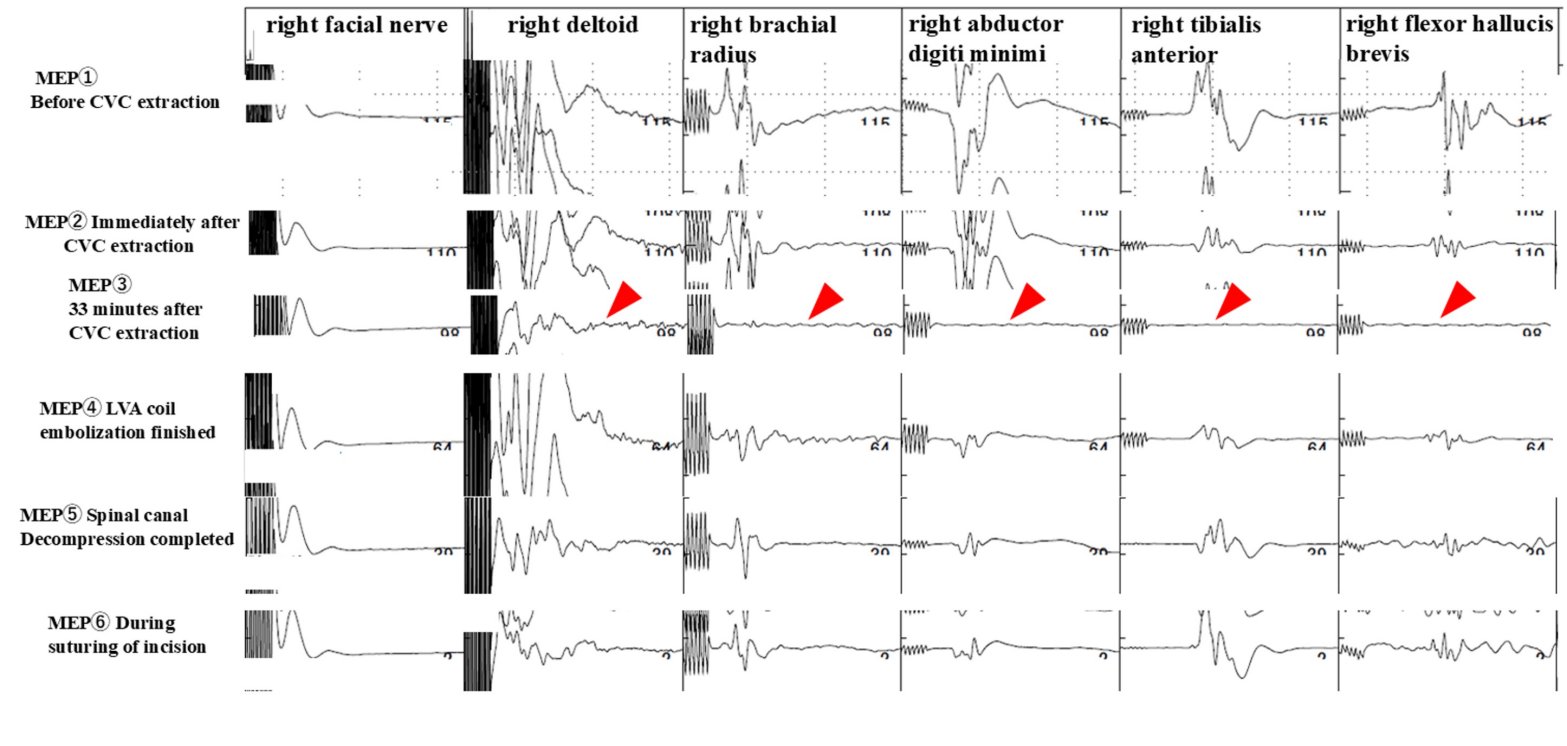
Motor-evoked potentials on the right side MEP: Motor-evoked potential; CVC: central venous catheter; LVA: left vertebral artery Changes in motor-evoked potentials were represented over time. The waveform attenuated with removal of the CVC and flattened after 33 minutes. The arrows emphasize where the waveform flattened out. After coil embolization of the left vertebral artery and emergency laminectomy, the waveform gradually increased and recovered

**Figure 4:**
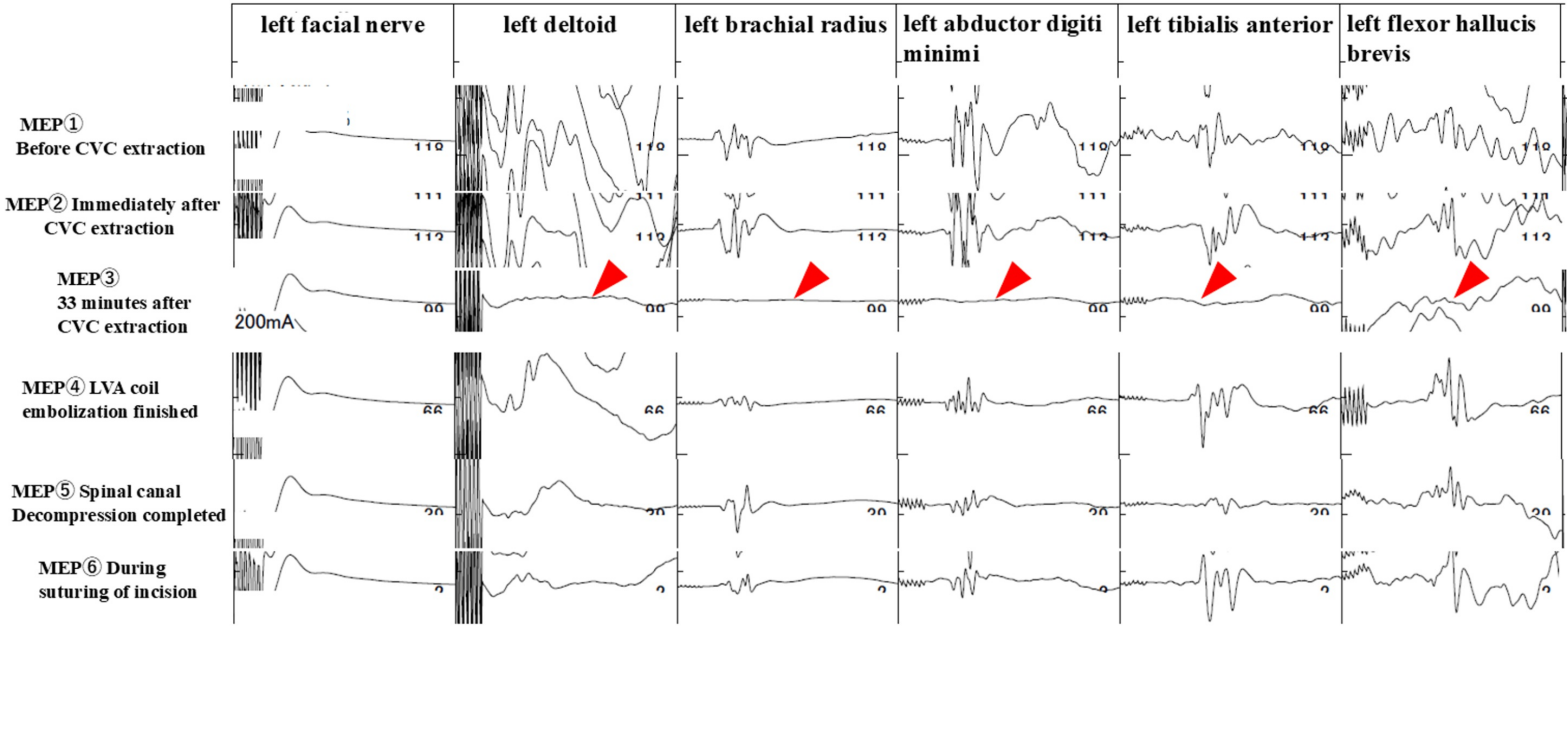
Motor-evoked potentials on the left side MEP: Motor-evoked potential; CVC: central venous catheter; LVA: left vertebral artery Changes in motor-evoked potentials were represented over time. The same course was observed with motor-evoked potentials on the right side. The arrows emphasize where the waveform flattened out

Angiography was performed by the neurosurgeons demonstrating no contrast leak from the patient’s left vertebral artery. The cardiovascular surgeon and otorhinolaryngologist made an incision at the site of the CVC insertion and dissected the tissue surrounding the CVC to confirm the injury of the left vertebral artery. The left vertebral artery was judged to be safe for removal of the CVC; therefore, the CVC was removed gently, after which there was arterial bleeding. The application of sutures effectively stopped the bleeding. However, the MEP waves gradually deteriorated. Consequently, angiography was performed again, which revealed injury of the left vertebral artery due to CVC insertion and bleeding from the artery into the epidural space. Blood pressure and heart rate, which were 122/58 mmHg and 63 beats/min, respectively, immediately after CVC removal increased to 162/74 mmHg and 99 beats/min, respectively, after 23 minutes. After 33 minutes of CVC removal, the MEP waveforms of all channels except the facial muscles completely disappeared. To control bleeding, neurosurgeons performed coil embolization at the site of the more proximal injury. After coil embolization, MEP waves were recognized again, although their amplitudes were one-third of those before the procedure, which is considered significant (Figures 3-4). Therefore, the orthopedic surgeons performed cervical laminectomy at the 6th cervical vertebra through the 1st thoracic vertebra. They reached the epidural space, revealing hematoma and continued bleeding from the epidural venous plexus. The epidural hematoma was removed, and a spinal canal decompression was performed, after which the MEP waves efficiently recovered. Coil embolization was performed in the central portion of the left vertebral artery; therefore, postoperative angiography was not performed due to the lack of an access route. Angiography from the right vertebral artery was not performed. The neurosurgeon decided that it was unnecessary because if the angiography resulted in poor flow in the peripheral left vertebral artery, it would have been difficult to treat. The total intraoperative blood loss was 830 mL, including 280 mL of external arterial bleeding at the time of CVC extraction and 550 mL of hematoma suctioned at the time of laminectomy.

Recognizing the risk of bleeding recurrence from the epidural venous plexus that could be triggered by body movements, continued sedation until the next day was planned. Accordingly, after the procedures, the patient was moved to the intensive care unit (ICU) without extubation. Immediately after ICU admission, the sedation medications were interrupted once to confirm that the patient could be slowly aroused, which was determined based on her ability to move her extremities and follow instructions. The patient was then placed under sedation. She was extubated 19 hours after returning to the ICU. She was able to walk on postoperative day 4, left the ICU on postoperative day 10, and was discharged on postoperative day 17. Because of hoarseness in the voice, follow-up with the otorhinolaryngologists was continued until eight months after the procedure. The patient did not experience other neurological complications.

## Discussion

There are a few reported cases of CVC straying into the epidural space. For example, Yokoyama et al. reported a similar case in which the CVC was carefully removed under fluoroscopic guidance. After a few minutes, the patient complained of severe back pain and mild paresis in his legs. Cervical laminectomy for spinal canal decompression was performed, after which the patient completely recovered [9]. They could immediately perform the required procedure because of the patient’s complaint. However, in our case, the need to make a deep incision to evaluate the vertebral artery required general anesthesia and did not allow for neurologic evaluation based on the patient’s complaint. Because our patient was under general anesthesia and could not provide feedback, we monitored the MEP waves. Immediately after CVC removal, arterial bleeding occurred, and MEP waves started to deteriorate. Based on the anesthetic record, although the volume of blood loss was 275 mL 30 minutes after CVC removal, the patient’s blood pressure increased. In addition, angiography revealed that the contrast leaked from the left vertebral artery into the epidural space. These findings indicated acute severe spinal canal compression, which might lead to complications. Notably, in this case, the earliest neurological complication was detected by observing MEP deterioration. In general, symptomatic epidural hematoma is treated with emergency laminectomy [10]. In this case, MEP deterioration continued even after coil embolization of the left vertebral artery although there were no other symptoms (because the patient was under general anesthesia); therefore, emergency cervical laminectomy was promptly performed. Intraoperative findings of cervical laminectomy showed a large amount of hematoma from the left vertebral artery bleeding and continued venous bleeding from the epidural venous plexus. The initial bleeding from the epidural venous plexus was triggered by the CVC insertion, and the compression of the epidural veins caused by the CVC temporarily achieved hemostasis. However, upon removal of CVC, rebleeding occurred because of the previously damaged veins.

**Figure 5:**
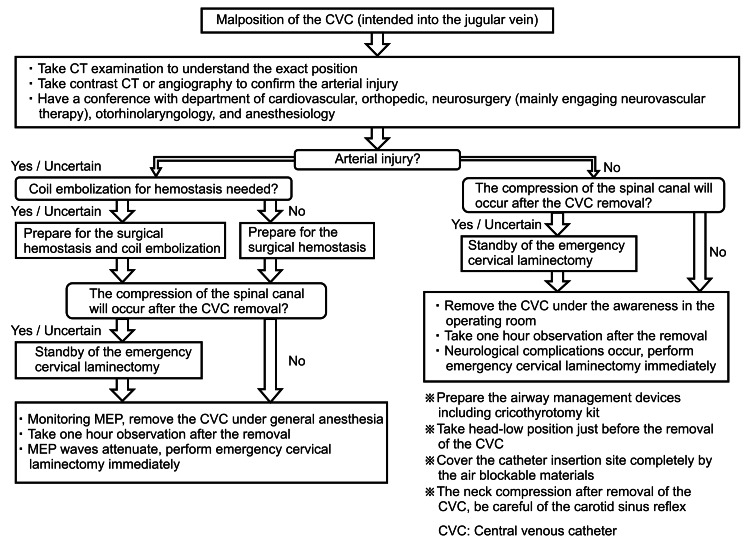
Case management and points considered during the removal of the malpositioned central venous catheter CVC: Central venous catheter; CT: computed tomography; MEP: motor-evoked potential The chart shows the considerations that were taken into account when determining the strategy in the present case

The findings of this case report directly indicate that bleeding from the epidural space or its vicinity may cause severe neurological complications. Therefore, if the CVC is malpositioned, a clinical decision on whether the removal of the CVC compresses the spinal canal or not is required. In cases where the CVC strays into the epidural space, as in our case, the epidural veins are damaged. Further, when the vertebral artery is injured, which causes bleeding that flows into the spinal canal, evaluation of arterial injury is necessary. If arterial injury is suspected, the use of the cutaneous approach may be difficult to stop. Therefore, coil embolization is necessary. In addition, the hematoma may compress the spinal canal, which may result in neurological complications; hence, neurological monitoring is required. Reddy et al. reported that it is not possible for the surgical team to recognize alterations in neurological function without the MEP. They also emphasized the importance of neurological monitoring [11]. Figure 5 presents the flowchart of the management of CVC malposition. There are two approaches for treating CVC malposition: the evaluation and treatment of arterial injury and the evaluation and treatment of neurological complications. Arterial injury may not be known until after the CVC is removed. Therefore, if it is uncertain whether the CVC injured the artery, the artery should be regarded as injured. In this case, management for arterial bleeding should be planned. Furthermore, if it is uncertain whether a superficial approach can stop the bleeding, transcatheter arterial embolization must be considered. Regarding the evaluation of neurological complications, it is possible to monitor neurological function in awake patients through feedback. However, patients who are under general anesthesia cannot voice their complaints; thus, MEP monitoring is needed. After the removal of the misplaced CVC, airway devices, including a cricothyrotomy kit, need to be prepared because of the risk of retropharyngeal hemorrhage, which may cause upper airway obstruction. Additionally, to take measures to early detect upper airway obstruction due to neck hemorrhage, patients should be observed for one hour after the removal of the CVC. This step is performed to early detect upper airway obstruction due to neck hemorrhage. Based on our hospital rule, patients who undergo neck surgeries are observed for one hour in the post-anesthesia care unit of the operating department. During CVC removal, patients should be positioned in a head-down tilt position, and an airtight dressing must be applied [8]. Additionally, a case was reported in which neck compression for hemostasis immediately after CVC removal caused cardiac arrest due to carotid sinus reflex [12]. Therefore, during CVC removal, hemostasis should be performed gently, and the patient should be carefully monitored.

## Conclusions

In this case, MEP monitoring was performed during the extraction of the misplaced CVC to treat severe neurological complications. When the CVC is malpositioned, it is crucial to monitor for any abnormal contact with the spinal canal, and MEP monitoring is essential during CVC removal under general anesthesia. 

We demonstrate that complications from CVC insertion can occur following the removal of the CVC when it is malpositioned. Therefore, it is essential to anticipate and prepare for potential complications to prevent severe outcomes.
